# Tailoring composite hydrogel performance *via* controlled integration of norbornene-functionalised Pluronic micelles

**DOI:** 10.1039/d5bm01434d

**Published:** 2025-12-03

**Authors:** Nicola Contessi Negrini, Hongning Sun, Adam D. Celiz

**Affiliations:** a Department of Bioengineering, Imperial College London London UK a.celiz@imperial.ac.uk; b The Francis Crick Institute London UK

## Abstract

Incorporating micelles into polymeric hydrogels offers a powerful route to combine the tuneable mechanical and structural properties of hydrogels with the precise drug-loading and release capabilities of nanocarriers. However, the method of micelle incorporation and its influence on hydrogel performance have yet to be studied in detail. Here, we present a modular strategy to tailor gelatin-norbornene hydrogels by integrating Pluronic® F127 micelles either physically or *via* covalent incorporation using norbornene-functionalised Pluronic (Pl_Nb). Pl_Nb was synthesised *via* Steglich esterification with >95% terminal functionalisation, forming stable, thermo-responsive micelles (2.5–15% w/v) with doxorubicin encapsulation efficiency of ∼80%, comparable to unmodified Pluronic. Micelles were either physically entrapped or chemically integrated into gelatin-norbornene networks *via* bioorthogonal thiol–ene crosslinking. The incorporation route dictated network mechanics and dynamics: chemical crosslinking conferred temperature-dependent behaviour and enhanced stress relaxation compared to physical crosslinking, whereas both incorporation routes reduced stiffness relative to neat hydrogels and slowed drug release compared to direct loading. All hydrogels were cytocompatible, and the released doxorubicin retained its bioactivity, reducing cancer cell viability. These findings establish micelle–hydrogel coupling as a versatile design approach for engineering biomaterials with potential in controlled therapeutic delivery and regenerative medicine.

## Abstract 2

Chemical modification of Pluronic micelles enables covalent crosslinking to hydrogels, contrasting physical entrapment for tunable biomaterial properties.

## Graphical abstract

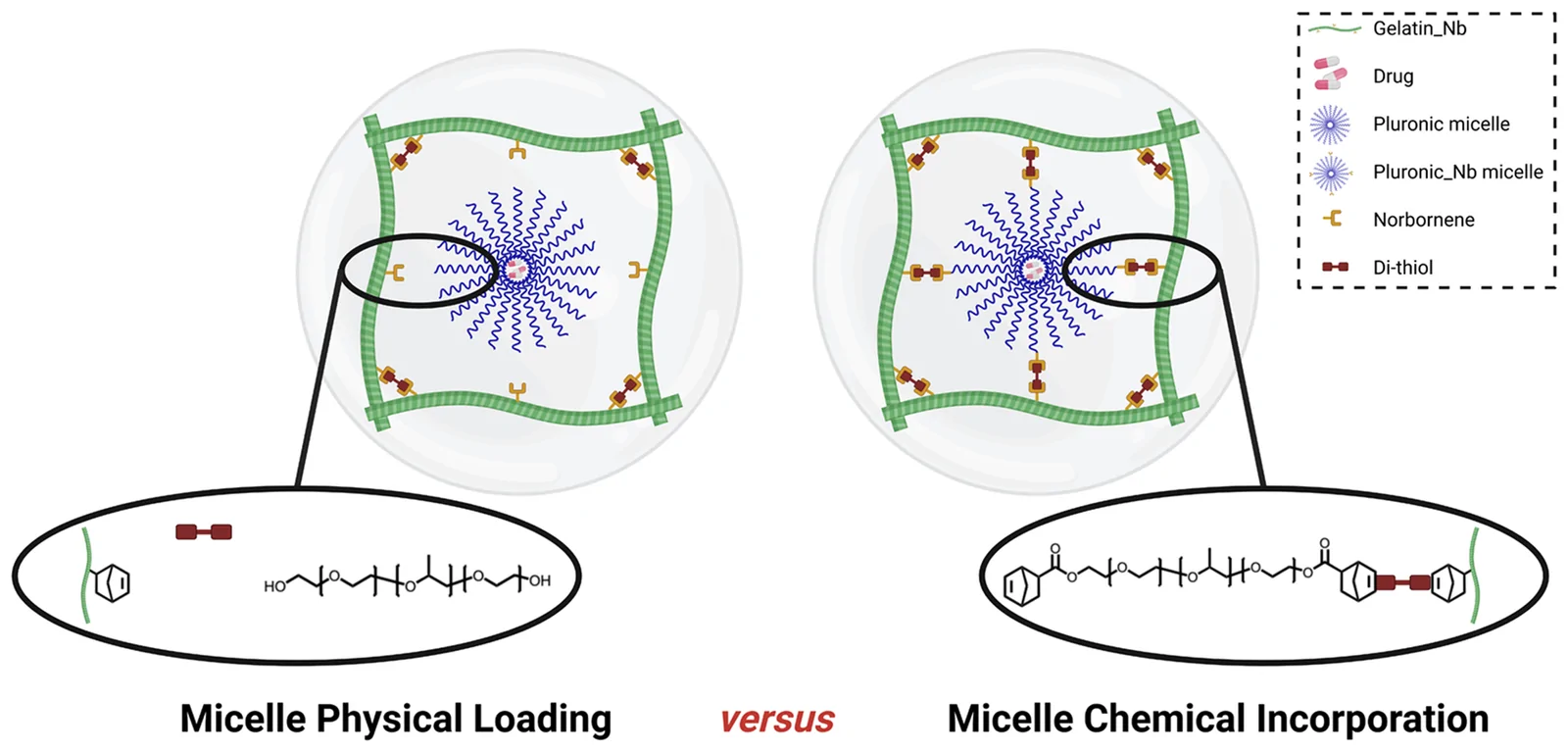

## 1. Introduction

The delivery of therapeutics is central to disease treatment^1^ and tissue engineering.^2^ As the field shifts from systemic to local administration of therapeutics, thanks to enhanced efficacy, reduced off-target effects, improved bioavailability, and the potential for lower dose administration, new technologies are required to act as delivery vehicles. Nanocarriers have gained significant attention for encapsulating hydrophobic drugs and modulating release profiles. Polymeric nanoparticles offer control over size, shape, surface properties, and responsiveness, enabling targeted delivery.^3^ However, nanoparticles alone may struggle to maintain sustained, local drug retention. Hydrogels, with tuneable mechanical and structural properties, complement nanoparticles by enabling localised release *via* diffusion or binding.^4^ Therefore, loading drug-loaded nanoparticles within hydrogels yields multifunctional composite systems adaptable for diverse applications, from cancer^5^ and chronic inflammation^6,7^ to tissue regeneration.^8^ More broadly, hydrogels can be engineered as hybrid nanocomposite systems^9^ by incorporating nanoscale components such as silica and carbon nanoparticles,^10^ cell-derived and biological nanoparticles,^11^ metal nanoparticles,^12^ and polymeric nanoparticles.^13^ These nanocomposites allow fine-tuning of hydrogel properties, including mechanical strength, porosity, swelling behaviour, and controlled release profiles, providing versatile platforms for biomedical applications. Pluronic micelles represent one class of such nanocomposite domains, offering dynamic and responsive features within the hydrogel network. Designing such systems requires understanding how parameters like crosslinking strategy, composition, and nanocomposite integration influence release kinetics, mechanics, and biocompatibility.^14^

Among the broad class of nanocarriers, Pluronic® (Pluronic; Pl) micelles have received substantial attention due to their self-assembling properties, amphiphilic structure, and chemical tunability.^15^ Pluronics are FDA-approved triblock copolymers composed of a hydrophobic poly(propylene oxide) (PPO) central block flanked by hydrophilic poly(ethylene oxide) (PEO) chains. In aqueous environments, they spontaneously form micelles with a PPO core and PEO corona once the concentration exceeds the critical micelle concentration and the temperature surpasses the lower critical solution temperature (LCST).^16^ These micelles have been extensively studied for solubilising hydrophobic drugs and improving their stability and bioavailability.^17,18^ Beyond their native form, Pluronic micelles have been chemically modified to increase stability, targeting capability, and responsiveness. For example, Pluronic has been conjugated to the photosensitizer chlorin e6 to improve tumour specificity and intracellular uptake for photodynamic therapy.^19^ In another application, Pluronic has been functionalised with biotin and rhodamine B to enable multifunctionality.^20,21^ Pluronic has also been conjugated to pyropheophorbide A for fluorescence-based early tumour imaging with improved biodistribution and safety profiles.^22^ Drug-loaded Pluronic micelles have also been engineered with folic acid to enhance solubility, tumour targeting, and therapeutic efficacy.^23,24^ Lipid–Pluronic hybrid micelles, incorporating phospholipids and maleimide-functionalized PEG chains, have been used to improve mucosal adhesion for ocular delivery.^25^ In gene delivery, Pluronic has been modified with disulfide linkers for intracellular siRNA release,^26^ or with pyridyl disulfide groups to enable thiol-exchange conjugation of targeting ligands such as transferrin.^27^

Pluronic micelles have also been incorporated into hydrogels *via* physical embedding or chemical crosslinking, depending on the desired application and performance.^28^ The most common method is physical incorporation, where pre-formed micelles are dispersed throughout the hydrogel matrix. Composite hydrogel networks with physically embedded micelles have been prepared for instance by combining Pluronic and diacrylate-functionalised PEGs enabling tuneable rheology and UV crosslinking, with micelle content influencing print fidelity.^29^ Additionally, hydroxypropyl cellulose/Pluronic micelle blends have shown promise for mucoadhesive scaffolds and intestinal drug delivery.^30^ In other systems, Pluronic/d-α-tocopheryl polyethylene glycol 1000 succinate (TPGS) mixed micelles were physically embedded into hydrogels for the topical delivery of glycyrrhizin acid, improving therapeutic outcomes in atopic dermatitis.^31^ Curcumin-loaded Pluronic micelles have also been incorporated into chitosan/polyethylene oxide nanofibers alongside zinc oxide nanoparticles to develop antibacterial wound dressings.^32^

In contrast, chemical incorporation involves covalently linking the micelles to the hydrogel network, either as part of the gelation process or as macro-crosslinkers. In some systems, the micelles themselves form the basis of the hydrogel by interlinking through covalent bonds.^33,34^ Examples of micelles chemically linked to a hydrogel network include Pluronic micelles grafted with benzaldehyde groups and crosslinked with gelatin *via* dynamic Schiff base reactions to create pH-responsive, injectable, and self-healing hydrogels.^35^ Alternatively, acrylated Pluronic has been photo-crosslinked with hyaluronic acid to produce stiff hydrogels suitable for hard tissue engineering.^36^ Another system used benzaldehyde-functionalised Pluronic and acylhydrazine-terminated PEG to form 3D-printable, highly stretchable hydrogels *via* reversible acylhydrazone bonds.^37^ Nanomicelle-crosslinked hydrogels, synthesized by photo-initiated polymerization of Pluronic diacrylate micelles with methacrylated hyaluronic acid, have demonstrated promising mechanical properties and low swelling for cartilage tissue repair.^38^

In this work, we developed a new platform in which Pluronic micelles can be incorporated into a gelatin-based polymer network *via* selective physical embedding or chemical crosslinking. We explore how the mode of micelle incorporation (*i.e.*, non-covalent dispersion *vs.* covalent integration) affects the structural, mechanical, and biological performance of the resulting hydrogels. For physical embedding, Pluronic® F127 (Pl) micelles are dispersed into thiol–ene crosslinked gelatin-norbornene hydrogels. For chemical incorporation, Pluronic® F127 is first functionalised with norbornene (Pl_Nb) to allow covalent integration through the thiol–ene chemistry during the gelatin-norbornene hydrogel crosslinking. We systematically compare the two strategies in terms of physicochemical properties, mechanical and rheological properties, and cytocompatibility. This work provides a modular framework for engineering tuneable micelle–hydrogel systems, with broad relevance for drug delivery and regenerative medicine.

## 2. Experimental

### 2.1. Materials

All reagents were purchased from Merck unless otherwise specified: Pluronic® F127 (Pl; *M*_w_ ≈ 12 600 g mol^−1^; PEO_100_–PPO_65_–PEO_100_), 5-norbornene-2-carboxylic acid (Nb; for Pluronic modification), 5-norbornene-2-methylamine (for gelatin modification), *N*,*N*′-dicyclohexylcarbodiimide (DCC), 4-(dimethylamino)pyridine (DMAP), dichloromethane (DCM), deuterated chloroform (chloroform-d) with 0.03% v/v tetramethylsilane (TMS), gelatin (X-Pure low-endotoxin type B from bovine bone, gel strength 240–270 g; Rousselot Biomedical), 2-(*N*-morpholino)ethanesulfonic acid hydrate (MES) buffer, *N*-hydroxysuccinimide (NHS), *N*-(3-(dimethylamino)-propyl)-*N*′-ethylcarbodiimide hydrochloride (EDC; Apollo Scientific), tetrahydrofuran (THF), 4′-hydroxyazobenzene-2-carboxylic acid (HABA), doxorubicin hydrochloride (doxorubicin, DOXO; Tokyo Chemical Industry UK Ltd), Methanol, Dulbecco's Phosphate Buffered Saline (DPBS), 2-hydroxy-4′-(2-hydroxyethoxy)-2-methylpropiophenone (Irgacure 2959), and PEG dithiol (PEG(SH)_2_, 3500 Da; JenKem®).

### 2.2. Cell cultures

Human dental pulp stem cells (hDPSCs; up to passage 6) and B16-F10 murine melanoma cells (up to passage 6) were cultured in Dulbecco's modified Eagle's medium (DMEM) supplemented with 10% v/v fetal bovine serum (FBS) and 1% v/v penicillin/streptomycin (P/S).

### 2.3. Synthesis of Pluronic-norbornene (Pl_Nb)

**Fig. 1:**
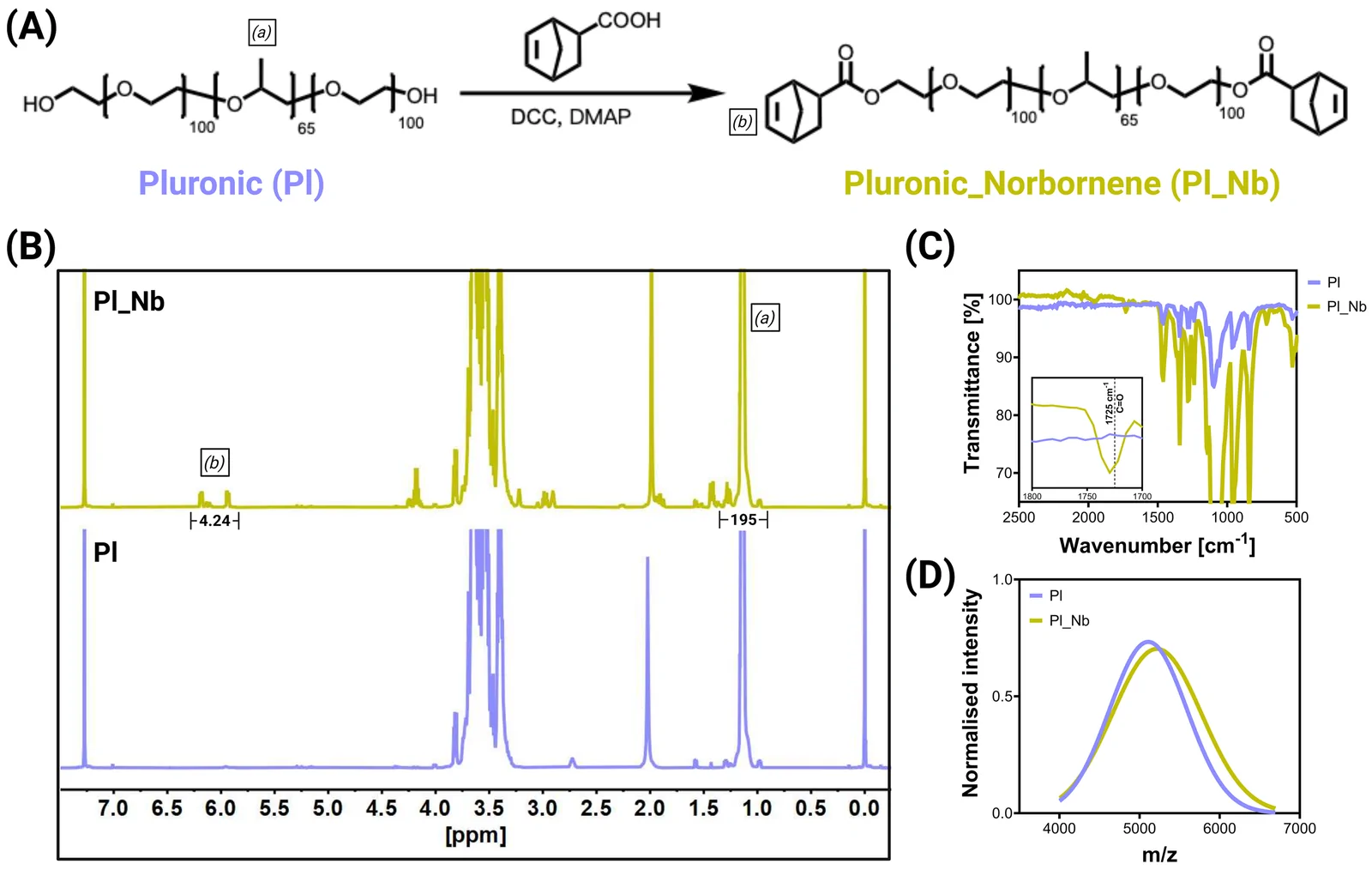
Functionalisation of Pluronic® F127 with norbornene *via* Steglich esterification. (A) Schematic illustration of the synthesis of norbornene-functionalised Pluronic® F127 (Pl_Nb) *via* Steglich esterification. (B) Representative ^1^H NMR spectra of unmodified Pluronic® F127 (Pl) and norbornene-functionalised Pluronic® F127 (Pl_Nb). (C) FTIR spectra of Pl and Pl_Nb, showing the characteristic C=O peak (1725 cm^−1^, inset) associated with norbornene incorporation. (D) MALDI TOF representative spectra of Pl and Pl_Nb.

Pl was functionalised with Nb by Steglich esterification (Fig. 1A). Reactions were conducted under nitrogen. Pl (10 g) was vacuum-dried overnight and dissolved in 130 mL anhydrous DCM. Nb (3 eq.) and DMAP (0.1 eq. previously dissolved in 10 mL DCM) were added dropwise and the reaction mixture was placed in an ice bath. DCC (1.1 eq. previously dissolved in 10 mL DCM) was then added dropwise and stirred at room temperature for 24 h. The product was then collected *via* rotary evaporator and dried under vacuum overnight. Then, the obtained solid product (Pl_Nb) was dissolved at 1% w/v in water, dialysed (molecular weight cut off MWCO = 3500 Da, 4 °C, 4 days), filtered (0.22 µm), and lyophilised.

The modification of Pl with Nb was verified *via*^1^H NMR. Samples (Pl and Pl_Nb) were prepared at 2.5% w/v in chloroform-d with 0.03% v/v TMS and tested using a Bruker Avance 500 MHz (256 scans, 5 s delay); the obtained spectra were analysed using MNova software (Mestrelab Research). The degree of modification (DoM) was calculated as (eqn (1)):1
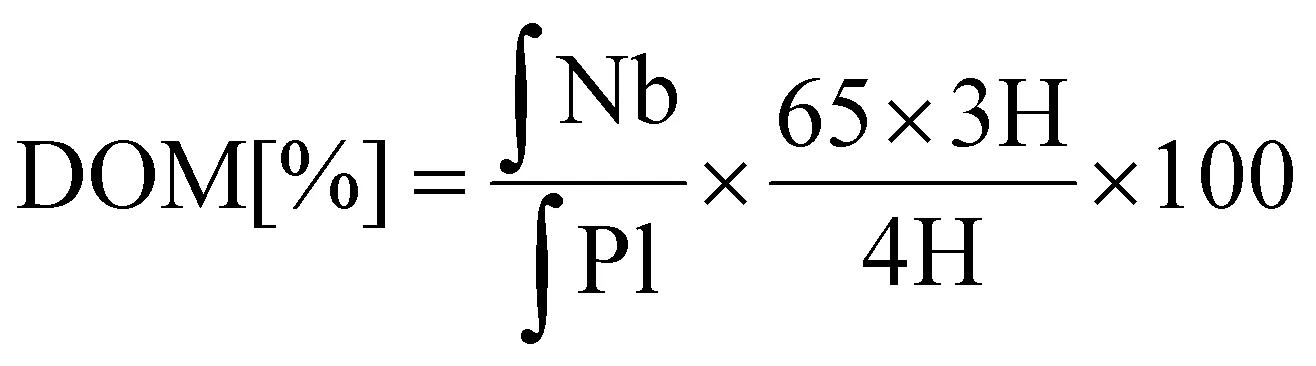
where 
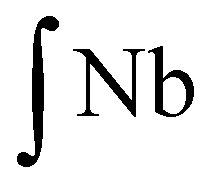
 is the signal detected for Nb (6.3–5.9 ppm per integrating for 4 protons), and 
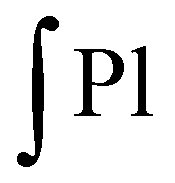
 is the internal reference signal of CH_3_ groups on Pl (1.4–0.9 ppm per integrating for 195 protons).

Fourier Transform Infrared Spectroscopy (FTRI; Cary 630) was performed in transmission mode to confirm ester bond formation, evidenced by the appearance of a peak at 1725 cm^−1^.

Matrix-Assisted Laser Desorption/Ionization Time-of-Flight Mass Spectrometry (MALDI-TOF) was used to assess the variation in molecular weight following Pl modification with Nb. HABA 10 mg mL^−1^ in THF was used as the matrix. Pl and Pl_Nb were dissolved in THF containing 0.1 M NaCl, used as a cationising agent. After mixing the samples with the matrix in a 1 : 1 v/v ratio, the solutions were loaded onto the MALDI target plate and analysed using a SHIMADZU MALDI-8030. Peaks were fitted using a Gaussian function, and the change in molecular weight was assessed based on the shift in the mean peak values.

### 2.4. Preparation and characterisation of Pl and Pl_Nb micelles

Micelles were prepared by thin-film hydration. Pl and Pl_Nb were dissolved in anhydrous methanol, then rotary-evaporated (Heidolph Hei-VAP) to form a thin film. Residual methanol was removed under vacuum overnight. The dry film was rehydrated with Milli-Q water at 2.5, 5, 10, or 15% w/v, sonicated for 5 min, and eventually filtered (diameter ∅ = 0.22 µm) to obtain the micelles.

The critical micelle concentration (CMC) was determined using pyrene fluorescence (wavelength *λ*_excitation_ = 320 nm, *λ*_emission_ = 373 nm) on a CLARIOstar Plus plate reader.

Micelle size was measured by dynamic light scattering (DLS, Malvern Panalytical Zetasizer Pro Blue); samples were equilibrated at 37 °C for 2 min before scanning (*n* = 3). Micelle morphology was visualised by transmission electron microscopy (TEM, JEOL JEM-2100F); 3 µL of solution was drop-cast on carbon-coated grids, blotted, air-dried, and imaged.

Temperature-dependent behaviour of the micelle suspensions was assessed by rheology (Netzsch Kinexus Ultra+, parallel plates, ∅ = 25 mm, gap = 1 mm, 1 Hz, 0.1% strain). Micelles (5% and 20% w/v) were analysed by temperature sweeps from 15 to 40 °C.

Micelle cytocompatibility was tested *via* a direct cytotoxicity test by seeding hDPSCs in 96-well plates at 1 × 10^4^ cells per well. When 70% confluent, cells were treated with 5% (w/v) Pl or Pl_Nb micelles, fresh medium (CTRL+), or medium pre-incubated with rubber band (CTRL−). Cell metabolic activity was assessed using 10% (v/v) alamarBlue™ (*λ*_excitation_ = 560 nm, *λ*_emission_ = 590 nm; CLARIOstar Plus; *n* = 6). Cell viability was expressed as percentage compared to the positive control.^39^

### 2.5. Composite hydrogel preparation and characterisation

Hydrogels were prepared using gelatin as polymer backbone. Gelatin was functionalised with 5-norbornene-2-methylamine by EDC/NHS coupling (Gel_Nb).^40^ Briefly, gelatin was dissolved at 1% w/v in 0.1 M MES buffer (pH 6) and reacted with 1 mmol g^−1^ gelatin of norbornene amine, EDC, and NHS (1 : 2 : 1 molar ratio) for 4 h at 37 °C. The solution was diluted with Milli-Q water (1 : 1), stirred for 30 min, dialysed (MWCO 3500 Da, 4 days), filtered, and lyophilised. Functionalisation was confirmed by ^1^H NMR as previously described.^41^

Hydrogels were prepared using Gel_Nb (10% w/v), Irgacure 2959 (0.5% w/v), and PEG dithiol (2 : 1 thiol : ene ratio) in PBS (GEL). To form composite hydrogels, Pl micelles or Pl_Nb micelles were added to the Gel_Nb precursors to obtain GEL_Pl and GEL_Pl_Nb precursors, respectively. Precursors were cast in PDMS moulds and photocrosslinked under UV light (40 mW cm^−2^, 180 s; Omnicure S1500) to form GEL, GEL_Pl, and GEL_Pl_Nb hydrogels (Fig. 3A).

Crosslinking kinetics were analysed by photo-rheology (Netzsch Kinexus Ultra+; parallel plates, ∅ = 25 mm, 0.5 Hz, 1% strain; *n* = 3). Hydrogel precursors were loaded on the quartz plate of the rheometer and the test started; during the test, the UV light was turned on to activate the crosslinking, and the evolution of the rheological properties was recorded.

Hydrogel swelling and stability were assessed over 7 days in PBS with 0.01% w/v sodium azide at 37 °C.^42^ Freshly prepared hydrogel samples (*n* = 5) were weighted (*w*_0_), lodged in 24-multiwell tissue culture polystyrene (TCPS), immersed in 1.5 mL PBS, stored at 37 °C, and weighted at established time points (*w*_*t*_) up to 14 days. The percentage weight variation Δ*w*[%] was calculated, at each time point *t* (eqn (2)):2
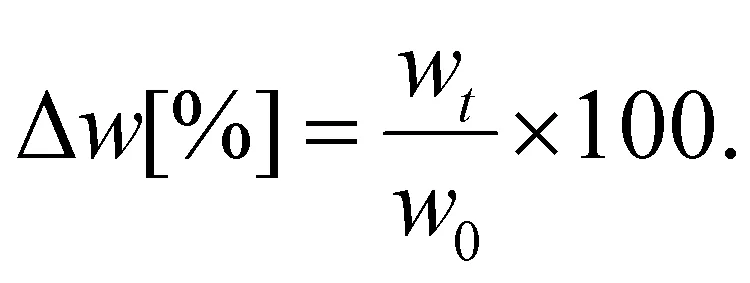


The mechanical properties of freshly made hydrogels (*n* = 3) were tested *via* indentation tests at room temperature. The test was performed using a Mach-1 Mechanical Testing System with a 2 mm diameter spherical probe. The indentation rate was 1 mm min^−1^, and the depth of indentation was 1 mm. The results were fitted by Hertz model, using the linear part of the indentation curve.

The temperature-responsive behaviour of crosslinked hydrogels (*n* = 4) was tested by temperature sweeps (from 5 to 55 °C at 1 °C min^−1^, parallel plates, ∅ = 25 mm, gap = 2.2 mm, 0.5 Hz, 0.1% strain). Stress relaxation was evaluated at 4 °C and 37 °C by applying a 10% strain to crosslinked discs and monitoring the stress decay over 5 minutes (*n* = 3). To analyse the rate and extent of stress relaxation, the normalised stress decay was fitted using a one-phase exponential decay model.

The cytocompatibility of the hydrogels was tested *via* direct cytocompatibility tests. GEL, GEL_Pl, and GEL_Pl_Nb hydrogels were prepared into 48-well plates (*n* = 8). Then, hDPSCs (15 000 cells per sample) were seeded and cultured for 7 days. The metabolic activity of cells was measured *via* alamarBlue™ and expressed as ratio percentage increase compared to day 1.

### 2.6. *In vitro* drug release and cell response

Doxorubicin (DOXO) was loaded into micelles by thin-film hydration. Pl and Pl_Nb were dissolved in methanol containing DOXO (1 mg per 50 mg polymer), rotary-evaporated, and vacuum-dried overnight. Films were rehydrated in Milli-Q water, centrifuged (4000 rpm, 1 h), filtered (0.22 µm) to remove unincorporated drug, and lyophilised.

To determine the Encapsulation Efficiency (EE) and the percentage Loading Capacity (LC), freeze-dried doxorubicin-loaded micelles (*n* = 3) were re-dissolved in DMF. The dissolved micelles were analysed by UV–vis spectroscopy (*λ* = 480 nm; CLARIOstar Plus) and the amount of DOXO was calculated using a calibration curve. The percentage EE and LC were calculated following eqn (3) and (4), respectively:^43^3
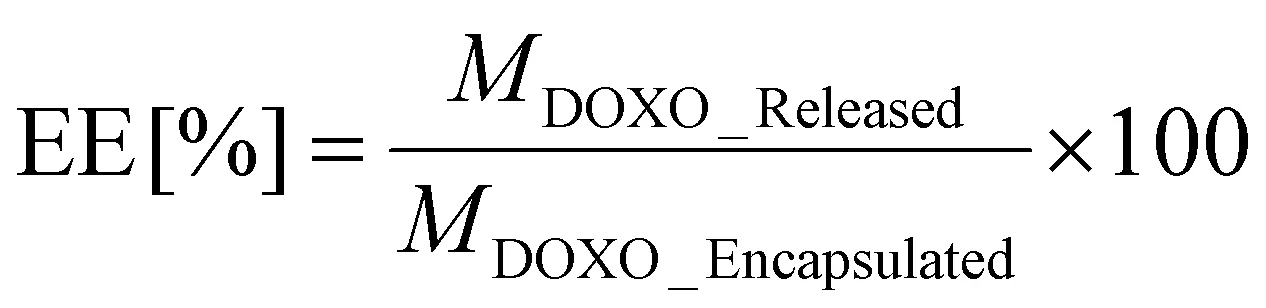
4
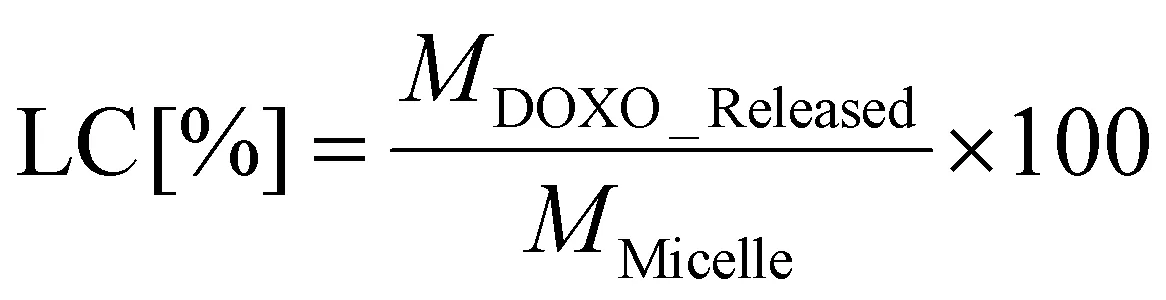
where *M*_DOXO_Released_ is the mass of doxorubicin released from the dissolved micelles, *M*_DOXO_Encapsulated_ is the mass of doxorubicin loaded in the micelles during their preparation, and *M*_Micelle_ is the mass of micelles tested.

The micelle size and zeta potential (ζ) were tested using a Malvern Zetasizer Pro. Lyophilised micelles were suspended in water, equilibrated at 37 °C for 10 min, and tested (*n* = 3).

Doxorubicin-loaded hydrogels were prepared using two approaches: (1) direct incorporation of the drug into gelatin hydrogels and (2) incorporation of doxorubicin-loaded Pluronic (Pl) micelles. For direct loading, doxorubicin was added to the Gel_Nb precursor either without micelles (GEL/DOXO) or with physically (GEL/DOXO_Pl) or chemically (GEL/DOXO_Pl_Nb) incorporated micelles. For micelle-mediated loading, doxorubicin was pre-encapsulated in Pl micelles (GEL_Pl/DOXO) or norbornene-functionalised Pl micelles (GEL_Pl_Nb/DOXO) prior to mixing with the Gel_Nb precursor. Hydrogels were then crosslinked as previously described. The release of doxorubicin from the hydrogels was investigated *in vitro*. Samples (*n* = 3) were incubated in PBS (2 mL, 37 °C), and 100 µL aliquots were collected at established timepoints over 3 days; the collected volume was replaced by fresh PBS at each time point. The cumulative release of doxorubicin was calculated measuring the concentration of released doxorubicin by UV–vis (*λ* = 480 nm; Nanodrop One, ThermoFisher) and normalised to the total amount of drug released at the final time point.

Cytotoxicity was assessed using B16-F10 cells. Cells were seeded at 2 × 10^4^ cells per cm^2^ in 96-well plates and cultured until 70% confluent. Then the culture medium was replaced by culture medium eluates obtained by incubation for 48 h with the following samples: gelatin hydrogels (GEL), Pluronic micelles (Pl), Pluronic_Norbornene micelles (Pl_Nb), gelatin hydrogels loaded with Pluronic micelles (GEL_Pl), gelatin hydrogels loaded with Pluronic_Norbornene micelles (GEL_Pl_Nb), and these samples loaded with doxorubicin (GEL/DOXO, Pl/DOXO, Pl_Nb/DOXO, GEL_Pl/DOXO, and GEL_Pl_Nb/DOXO). As controls, we used complete medium incubated for 48 h with no samples (CTRL), and doxorubicin dissolved 2.5 µg mL^−1^ in culture medium and incubated for 48 h (CTRL/DOXO). After 24 h incubation with sample eluates, cell metabolic activity was measured using 10% v/v alamarBlue™ (*n* = 6) and compared to the metabolic activity of cells cultured in fresh medium to evaluate the percentage cells viability (eqn (5)):5
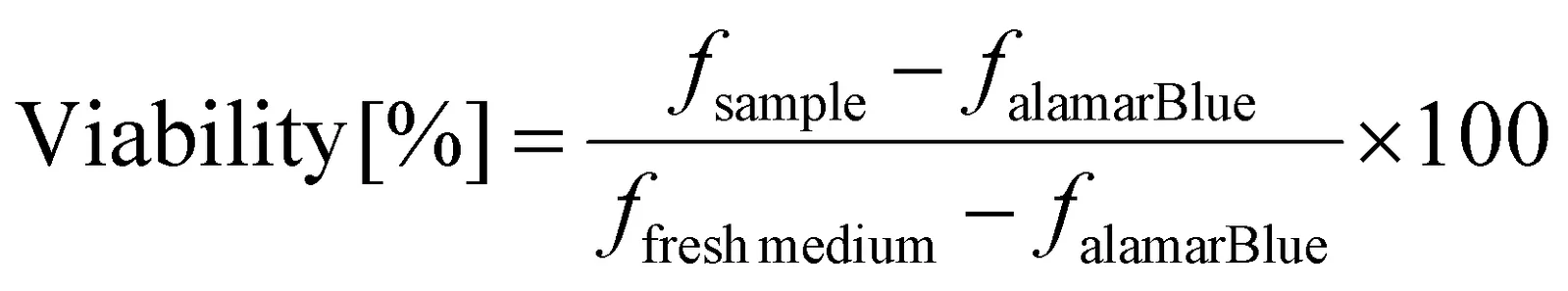
where *f*_sample_ is the fluorescence intensity measured for cells in contact with the samples, *f*_fresh medium_ the fluorescence intensity measured for cells cultured in fresh medium, and *f*_alamarBlue_ is the background signal of the alamarBlue solution.

### 2.7. Statistical analysis

Data are presented as mean ± standard deviation. Data normality was assessed using the Shapiro–Wilk test. One-way ANOVA with Tukey's *post hoc* test was used to evaluate statistical significance (*p* < 0.05, *p* < 0.01, *p* < 0.001, *p* < 0.0001). GraphPad Prism v10.2.1 was used for all analyses. Illustrations were created using BioRender.com.

## 3. Results and discussion

### 3.1. Synthesis of Pluronic_Norbornene (Pl_Nb)

We modified Pluronic® F127 (Pl) with norbornene (Nb) *via* Steglich esterification, covalently binding the carboxylic acid groups of Nb to the terminal hydroxyl groups of Pl (Fig. 1A). Pluronic® F127 was selected due to its widespread use in the formation of drug-loaded micelles, as well as its versatility for chemical modification, biocompatibility, thermo-responsive behaviour, and ability to assemble into macro- and nanostructures. Additionally, these self-assembled nanostructures can be embedded into hydrogels to generate composite biomaterials.^44^ We adapted existing protocols describing the functionalisation of poloxamers *via* Steglich esterification to optimise the conjugation of Nb to Pl.^45,46^ Specifically, the terminal hydroxyl groups of the triblock copolymer Pluronic® F127 were reacted with the carboxylic acid group of Nb using DCC/DMAP to yield norbornene-functionalised Pluronic (Pl_Nb).

The mass yield of the functionalisation reaction was approximately 80%. Successful incorporation of norbornene was confirmed by ^1^H NMR spectroscopy (Fig. 1B): the characteristic norbornene signals appeared at 6.5–6.0 ppm in the spectrum of Pl_Nb (peak (b), Fig. 1B top), which were absent in the unmodified Pl spectrum (Fig. 1B bottom). Importantly, the signal corresponding to the methyl groups of Pl (peak (a), Fig. 1B) remained unchanged in both spectra, indicating that the polymer backbone was not affected during terminal group modification.^47^ The degree of modification (DOM), calculated as the percentage of Pl hydroxyl groups functionalised with Nb, was 95 ± 4%, based on four independent syntheses. The ratio between the intensity of the methyl peak and the intensity of the Nb peak of Pl_Nb was 46.5 (theoretical ratio (65 × 3) : 4 = 48.7), indicating near-complete functionalisation of Pl hydroxyl groups with Nb. The DOM we achieved is comparable to reported values for poloxamers functionalised *via* hydroxyl group modification in the literature.^48,49^ FTIR spectroscopy further confirmed the successful conjugation of Nb to Pl, with the appearance of a new C=O stretching peak at 1725 cm^−1^ in the Pl_Nb spectrum, which is absent in the unmodified Pl spectrum (Fig. 1C). MALDI-TOF analysis also supported the successful modification, revealing a mass shift consistent with the expected norbornene functionalisation (Fig. 1D and Fig. S1).

The confirmed presence of Nb on Pl enables downstream bioorthogonal thiol–ene crosslinking, allowing the formation of Pl_Nb micelles that can be chemically crosslinked into hydrogel networks. This contrasts with unmodified Pl, which can only be physically embedded. These two strategies (*i.e.*, chemical *versus* physical incorporation) form the basis of the next sections, where we explore their implications for micelle behaviour and hydrogel composite design for drug delivery and their implications in biomaterial properties.^28^

### 3.2. Characterisation of Pluronic_Nb micelles

**Fig. 2:**
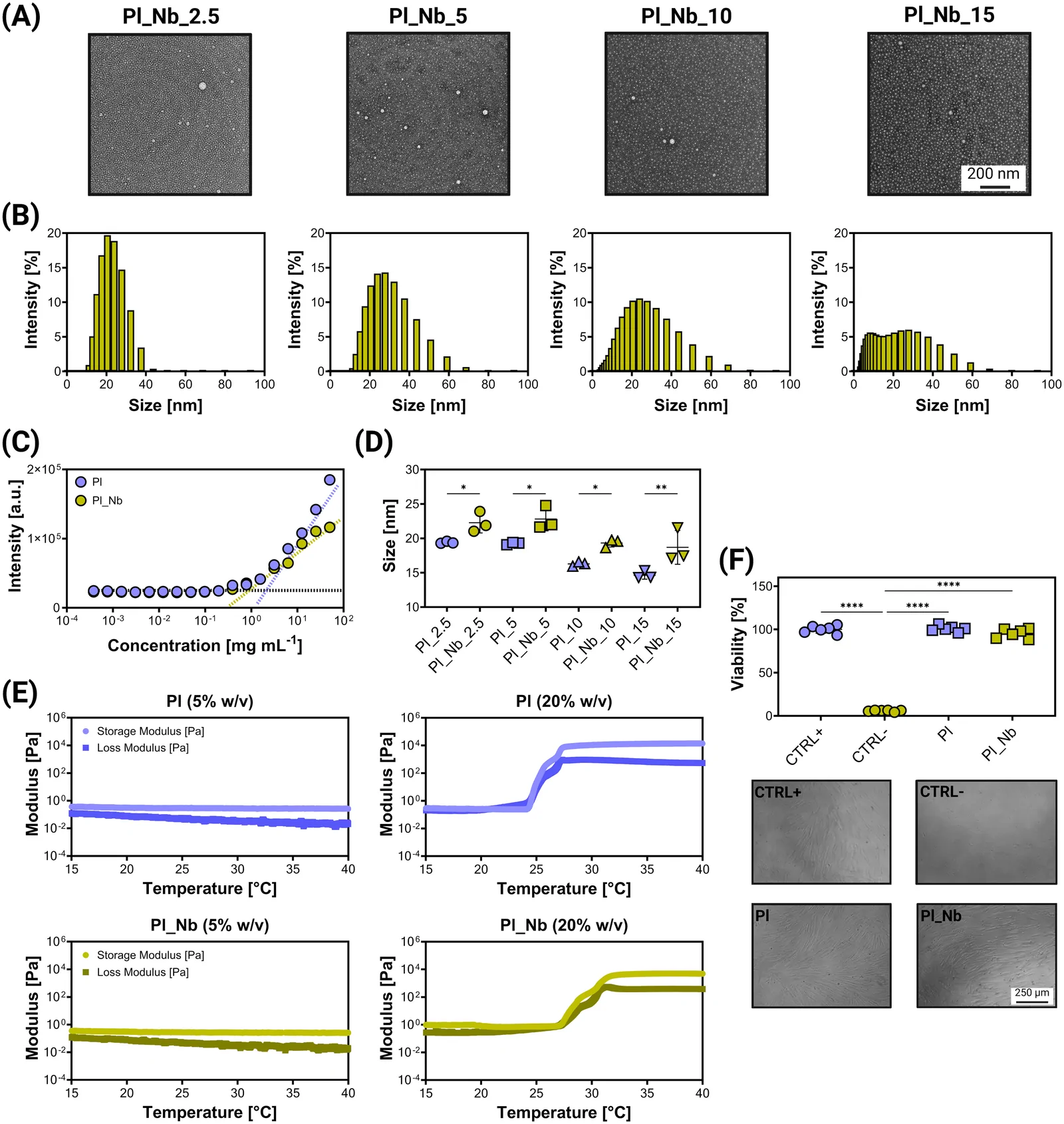
Characterisation of Pluronic_Norbornene (Pl_Nb) and Pluronic (Pl) micelles. (A) Representative TEM images of micelles prepared at varying concentrations (2.5, 5, 10, and 15% w/v) using the thin-film hydration method (scale bar = 200 nm). (B) Size distribution of Pl_Nb micelles prepared at different concentrations. (C) Critical micelle concentration (CMC) of Pl and Pl_Nb determined *via* pyrene fluorescence assay. (D) Comparison of the average size of micelles prepared with Pl and Pl_Nb across increasing concentrations (*n* = 3). (E) Thermo-responsive behaviour of Pl and Pl_Nb solutions at 5% and 20% w/v. (F) *In vitro* cytocompatibility of Pl and Pl_Nb micelles. Percentage cell viability following culture with control medium (CTRL+), medium incubated with a rubber band (CTRL−), and media containing Pl or Pl_Nb micelles (*n* = 6). Representative phase-contrast images of cells under each condition (scale bar = 250 µm).

We then investigated the ability of Pl_Nb to form micelles across a range of concentrations (2.5, 5, 10, and 15% w/v; referred to as Pl_Nb_2.5, Pl_Nb_5, Pl_Nb_10, and Pl_Nb_15, respectively), with the aim of identifying suitable concentrations for subsequent hydrogel precursor preparation and hydrogel formation. TEM micrographs revealed the presence of Pl_Nb micelles at all tested concentrations (Fig. 2A). Nanometric micelle formation was further confirmed by DLS, which showed the micelle size distribution in the nanometre range (Fig. 2B). The average diameter and polydispersity index (PDI) for each condition are summarised in Table S1. The micelle sizes ranged from 18 to 26 nm, which is consistent with previously reported sizes for chemically modified poloxamer micelles.^50^ A decrease in micelle size was observed when comparing Pl_Nb_10 and Pl_Nb_15 *vs.* Pl_Nb_2.5 and Pl_Nb_5 (Table S1). At higher concentrations, micelles form more readily, leading to crowding and closer packing, which can limit individual micelle size growth.^51^ This interpretation is supported by an increase in PDI at higher Pl_Nb concentrations (Table S1). As concentration increases, micelle formation becomes more dynamic, resulting in a mixture of populations and, potentially, the formation of micelle–micelle aggregates. This leads to a broader size distribution, a phenomenon also reported for unmodified Pl micelles.^52^

Micelle formation at the tested concentrations was further confirmed by determining the critical micelle concentration (CMC), which was found to be lower than all concentrations tested for micelle formation (Fig. 2C). For unmodified Pl, the CMC was 1.21 mg mL^−1^, consistent with the reported range of 0.2–1.5 mg mL^−1^ in the literature.^53^ After modification with Nb, the CMC of Pl_Nb decreased to 0.73 mg mL^−1^, indicating enhanced micelle stability. This is likely because the Nb modification decreases the hydrophile-lipophile balance (HLB) by introducing a more hydrophobic moiety; a lower HLB value can contribute to a reduced CMC.^54^

When comparing micelle sizes formed by Pl and Pl_Nb at the same concentrations, Pl_Nb micelles were relatively larger (Fig. 2D). This increase in size may be attributed to the presence of norbornene moieties at the polymer termini, which can increase the overall hydrodynamic diameter. Similar increases have been observed in micelles formed from end-modified polymers or block copolymers.^46,55^

Pl solutions are known to exhibit reverse thermo-responsive behaviour in aqueous environments, undergoing a sol–gel transition (*T*_sol–gel_) at characteristic temperatures. We investigated whether Pl_Nb retained this behaviour and observed that both Pl and Pl_Nb showed comparable thermo-responsive profiles (Fig. 2E). At 5% w/v, neither solution exhibited a significant thermo-responsive transition, likely due to insufficient micelle–micelle interactions to support network formation.^56^ In contrast, at 20% w/v, both solutions displayed a clear sol–gel transition, with increases in both storage modulus (G′) and loss modulus (G″) above *T*_sol–gel_.^57^ Notably, Pl_Nb displayed a slightly higher transition temperature than Pl, consistent with a shift in the effective LCST; yet, the transition remained below physiological temperature, ensuring gel formation under body-relevant conditions. This behaviour was also confirmed qualitatively *via* vial inversion tests, showing that both Pl and Pl_Nb solutions formed self-supporting gels at 37 °C (Fig. S2).

Finally, we assessed the cytocompatibility of Pl and Pl_Nb micelles *via* a direct *in vitro* cytotoxicity assay using human dental pulp stem cells (hDPSCs) as a model (Fig. 2F). Micelles were dissolved in cell culture medium and used to culture cells directly. When cells were cultured with either Pl or Pl_Nb micelles dispersed in the culture medium, cell viability remained above 70%, the threshold for cytotoxicity, and was comparable to that observed in the complete medium positive control (CTRL+). These results were supported by the healthy, elongated morphology of the cultured hDPSCs.

### 3.3. Pluronic micelle loaded gelatin hydrogels *via* physical and chemical incorporation

**Fig. 3:**
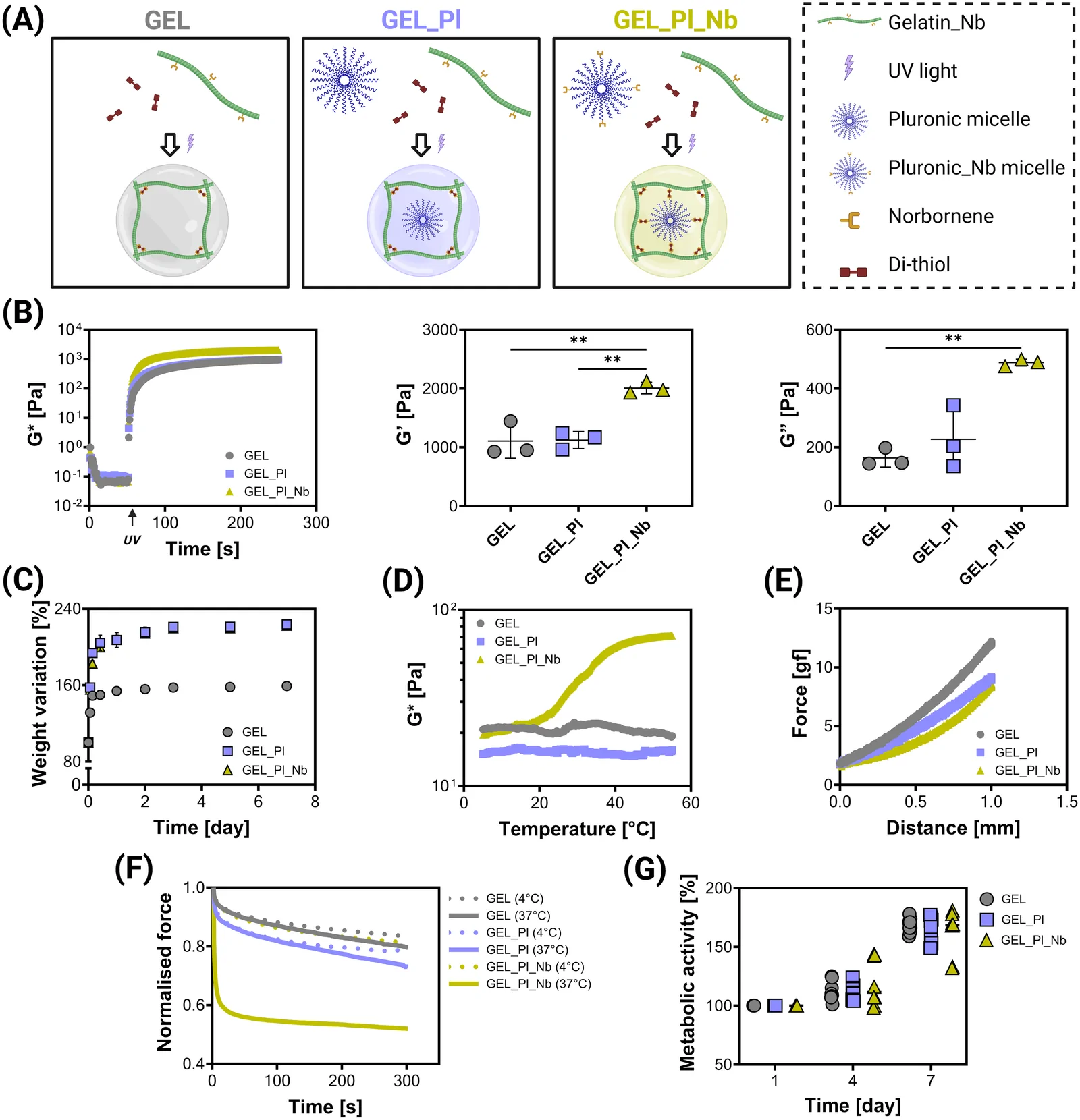
Preparation and characterisation of Pluronic micelle-laden gelatin hydrogels. (A) Schematic representation of hydrogel formation *via* thiol–ene crosslinking under UV irradiation: neat gelatin hydrogels (GEL), gelatin hydrogels loaded with Pluronic micelles (GEL_Pl), and gelatin hydrogels loaded with Pluronic_Norbornene micelles (GEL_Pl_Nb). (B) Representative rheological time sweep showing gelation of hydrogels after exposure to UV light; storage modulus G′ and loss modulus G″ at crosslinking plateau (*n* = 3). (C) Weight variation of hydrogels during incubation in PBS at 37 °C (*n* = 5). (D) Thermo-responsive properties of the crosslinked hydrogels. (E) Representative indentation curves. (F) Stress relaxation behaviour of crosslinked hydrogels during indentation. (G) Metabolic activity of hDPSCs cultured on the surface of GEL, GEL_Pl, and GEL_Pl_Nb hydrogels expressed as percentage increase to day 1 (*n* = 8).

We then investigated the influence of physical and chemical incorporation of micelles into gelatin-norbornene (Gel_Nb) hydrogels by loading Pl and Pl_Nb micelles, respectively (Fig. 3A). We selected gelatin as the hydrogel polymeric backbone due to its biocompatibility, presence of cell adhesive motifs (RGD sequences), and versatility for functionalisation and fabrication.^58,59^ We modified gelatin with norbornene to enable thiol–ene crosslinking in the presence of a dithiol crosslinker, photoinitiator, and UV light. This crosslinking chemistry has been widely used for cell microencapsulation,^60^ bioprinting,^61^ and tissue engineering scaffolds.^62^ We functionalised gelatin following a previous protocol,^41^ and obtained a degree of modification of approximately 10%, calculated as the percentage of carboxylic groups of gelatin decorated with Nb from the ^1^H NMR spectra (Fig. S3).

We then used the Gel_Nb precursors to prepare control gelatin hydrogels (GEL) without any micelles. To test the physical and chemical incorporation of micelles in the hydrogels, we loaded Pl micelles and Pl_Nb micelles into the Gel_Nb hydrogel precursor. Specifically, when Pl micelles are added to the Gel_Nb precursor, the bioorthogonal thiol–ene reaction between norbornene and thiol groups does not involve the micelles, resulting in their physical entrapment within the hydrogel network. In contrast, when Pl_Nb micelles are added, the norbornene groups present on the micelles can participate in the thiol–ene-based crosslinking, chemically binding the Pl_Nb micelles to the Gel_Nb polymer network and integrating them structurally into the hydrogel.

All the hydrogels (GEL, GEL_Pl, and GEL_Pl_Nb) crosslinked after exposure to UV light, as shown by the steep increase of the rheological complex modulus G* after irradiation (Fig. 3B). After crosslinking, the GEL_Pl_Nb hydrogels showed higher storage modulus G′ and loss modulus G″ compared to the other formulations, due to the presence of additional norbornene moieties that can engage in further crosslinking reactions, increasing network density.

The crosslinked hydrogels increased in weight after immersion in PBS at 37 °C and reached a swelling plateau, indicating effective crosslinking across all conditions and the formation of a gelatin polymer network that does not dissolve in water (Fig. S4). The stability and crosslinking of the hydrogels was also confirmed macroscopically by the maintenance of the hydrogel cylindrical shapes before and after swelling (Fig. 3C). Compared to GEL hydrogels, GEL_Pl and GEL_Pl_Nb hydrogels absorbed more water and swelled more, indicating a looser gelatin hydrogel network. In literature, the influence of micelle incorporation on hydrogel swelling is highly system-dependent, with reports of both reduced^63^ and enhanced^64^ swelling after micelle addition. Here, we hypothesise that the increased swelling observed in our micelle-containing hydrogels (both physically and chemically incorporated) arises from the micellar domains altering the network architecture and crosslinking density, introducing additional free volume and heterogeneity that promote water uptake and increase overall hydrogel swelling.

The influence of chemically crosslinked Pl_Nb micelles into the gelatin hydrogel polymer network was particularly visible when investigating the hydrogel mechano-rheological properties. First, we investigated the temperature responsiveness of the hydrogels (Fig. 3D and Fig. S5). The crosslinked gelatin sample, GEL, did not respond to variations in temperature due to the covalent thiol–ene bonds formed between gelatin polymer chains during crosslinking, as shown in literature by other chemically crosslinked gelatin hydrogels.^65^ The presence of physically embedded Pl micelles in GEL_Pl did not influence thermo-responsive behaviour. In contrast, a three-fold increase (Fig. S6) in rheological properties with increasing temperature was observed for hydrogels in which Pl_Nb micelles were chemically incorporated into the network (GEL_Pl_Nb). This reverse thermo-responsive behaviour, characteristic of Pluronic, arises from its increased viscosity at higher temperatures.^66,67^ Importantly, DLS measurements confirmed that both Pl and Pl_Nb micelles retained their micellar size across with increasing temperature (from 5 to 55 °C, Fig. S7), indicating that micelles are preserved within the tested temperature range.

The presence of micelles within the hydrogels decreased the hydrogel mechanical properties when tested *via* indentation (Fig. 3E). The instantaneous modulus (Fig. S8) of the neat gelatin hydrogels (Modulus_GEL_ = 4.94 ± 0.26 kPa) was reduced when Pl micelles were physically embedded (Modulus_GEL_Pl_ = 3.33 ± 0.43 kPa) and further reduced when Pl_Nb micelles were chemically incorporated into the hydrogel network (Modulus_GEL_Pl_Nb_ = 2.03 ± 0.14 kPa). Previous studies have shown that micelles can interfere with hydrogel crosslinking due to steric hindrance or competition for crosslinking sites,^68^ while others have shown that physical incorporation does not affect stiffness, whereas chemical incorporation can enhance mechanical properties through increased crosslink density.^69,70^ Here, we hypothesise that physical incorporation of micelles may interfere with Gel_Nb network formation, while chemical incorporation may replace covalent crosslinks between gelatin chains with micellar hydrophilic–hydrophobic domains that soften the network and reduce bulk stiffness. Notably, indentation tests at room temperature revealed this decrease in stiffness, whereas rheological measurements at higher temperatures show increased moduli due to the thermo-responsive behaviour of the chemically incorporated Pl_Nb micelles.

The chemical incorporation of Pl_Nb micelles also influenced the stress relaxation behaviour of the hydrogels (Fig. 3F). GEL hydrogels displayed predominantly elastic behaviour, with no variation in response with temperature, confirming the presence of a covalent crosslinked network and lack of thermo-responsiveness, consistent with the rheological temperature sweep. Similarly, physically incorporated Pl micelles (GEL_Pl) did not significantly affect temperature-dependent stress relaxation, although these hydrogels relaxed more after indentation compared to GEL. In contrast, chemically incorporated Pl_Nb micelles (GEL_Pl_Nb) showed a pronounced stress relaxation response that was also temperature dependent. This confirms the contribution of chemical incorporation of micelles to the hydrogel network dynamics and highlights their impact on temperature-mediated energy dissipation. When tested at physiological temperature, the GEL_Pl_Nb hydrogels, compared to other formulations, exhibited faster relaxation times (Fig. S9A), indicating that chemically incorporated micelles promote energy dissipation and stress relaxation. This is further confirmed by the lower normalised stress at plateau (Fig. S9B). We next examined whether the stress relaxation behaviour could be tuned by varying the ratio of chemically incorporated Pl_Nb micelles to physically incorporated Pl micelles during hydrogel preparation (*i.e.*, Pl_Nb = 0, 30, 60, and 100%; Fig. S10A). Increasing the proportion of Pl_Nb micelles within the network progressively enhanced stress relaxation, as evidenced by shorter relaxation times (Fig. S10B) and lower normalised stress at plateau (Fig. S10C). These findings demonstrate that covalent integration of micelles provides a controllable handle to modulate the viscoelastic response of the material. Such tunability of stress relaxation represents an important design feature with implications for future translational applications, as matrix viscoelasticity can influence both drug release kinetics^71^ and cell–matrix mechanical interactions.^72^ Future work will exploit this property to investigate how stress relaxation governs molecular diffusion and cellular responses within these hydrogels.

Finally, all the hydrogel formulations were cytocompatible as shown by the increase in the metabolic activity of hDPSCs cultured on the surface of the hydrogels (Fig. 3G), showing the possibility of cells to adhere to the gelatin polymer network independently from the presence of Pluronic micelles, either physically or chemically incorporated.

### 3.4. Drug release

We next investigated the potential of Pluronic micelles to encapsulate a chemotherapeutic drug and serve as delivery vehicles within hydrogels. Doxorubicin (DOXO) was selected as a model drug and successfully loaded into both Pl and Pl_Nb micelles. The encapsulation efficiency (EE%) and loading capacity (LC%) of DOXO were comparable between Pl and Pl_Nb micelles (Table 1), indicating that Nb modification does not impair drug loading. These values were in line with previously reported data for doxorubicin-loaded Pluronic micelles.^73^

**Table 1:** Encapsulation efficiency (EE%) and loading capacity (LC%) of doxorubicin in Pluronic (Pl) and Pluronic_Norbornene (Pl_Nb) micelles (*n* = 3)

	Pl micelles	Pl_Nb micelles
EE [%]	83.2 ± 3.0	81.0 ± 8.0
LC [%]	1.7 ± 0.1	1.5 ± 0.2

**Fig. 4:**
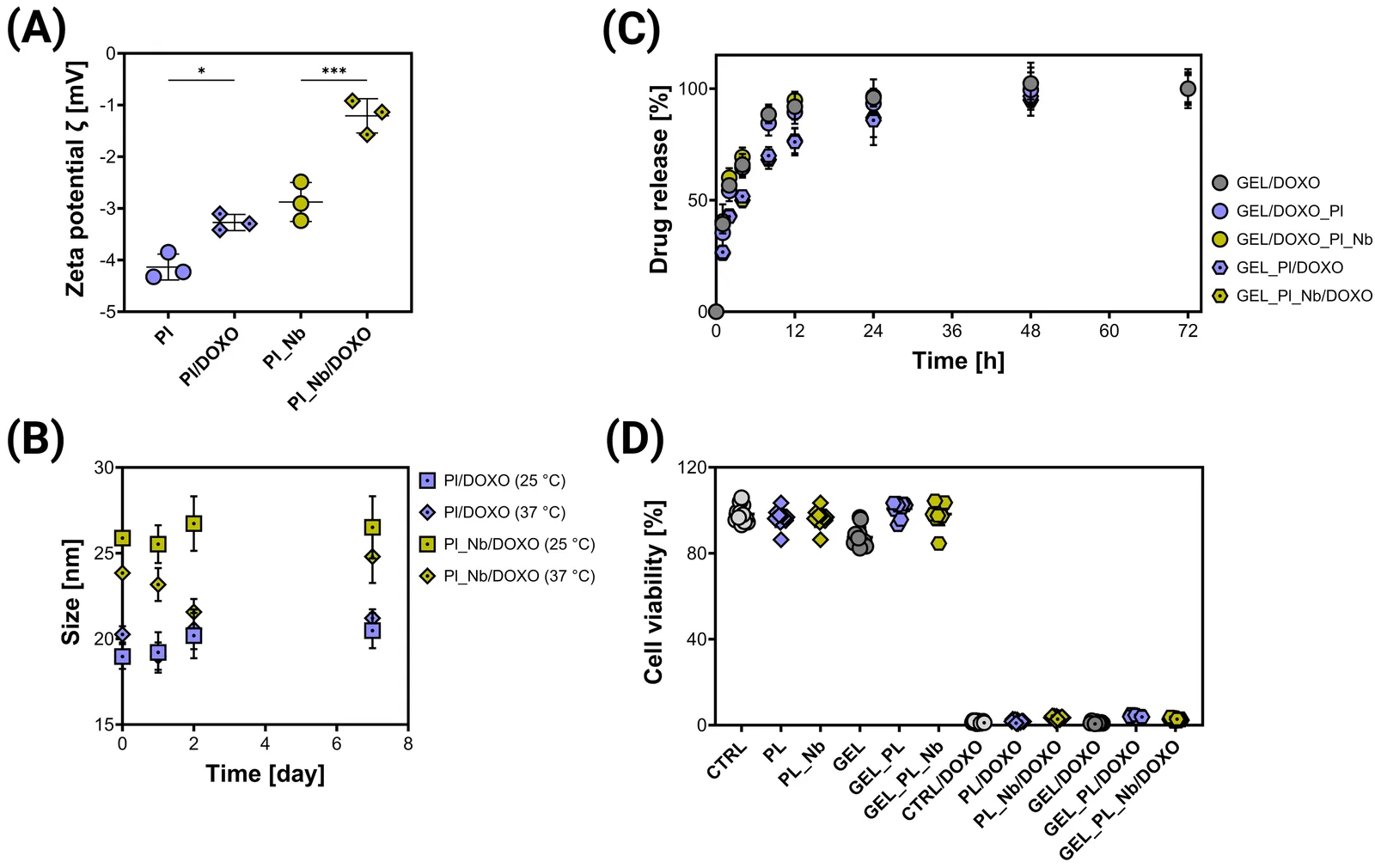
Characterisation of doxorubicin loading and release from micelles and micelle-laden hydrogels. (A) Zeta potential of Pluronic (Pl) and Pluronic_Norbornene (Pl_Nb) micelles (*n* = 3), before and after doxorubicin loading (Pl/DOXO and Pl_Nb/DOXO). (B) Size stability of doxorubicin-loaded micelles stored at 25 °C and 37 °C in PBS. (C) *In vitro* release profile of doxorubicin from gelatin hydrogels directly loaded with the drug (GEL/DOXO, GEL/DOXO_Pl, and GEL/DOXO_Pl_Nb), from gelatin hydrogels incorporating doxorubicin-loaded Pl micelles (GEL_Pl/DOXO), and from gelatin hydrogels incorporating and covalently crosslinked with doxorubicin-loaded Pl_Nb micelles (GEL_Pl_Nb/DOXO); *n* = 3. (D) Viability of B16F10 cells cultured in fresh medium (CTRL), medium with free doxorubicin (CTRL/DOXO), and supernatants collected from unloaded samples (GEL, Pl, Pl_Nb, GEL_Pl, GEL_Pl_Nb) and corresponding doxorubicin-loaded samples (GEL/DOXO, Pl/DOXO, Pl_Nb/DOXO, GEL_Pl/DOXO, GEL_Pl_Nb/DOXO; *n* = 6).

To confirm successful encapsulation, we measured the zeta potential ζ and hydrodynamic diameter of unloaded and doxorubicin-loaded micelles. Both Pl and Pl_Nb micelles exhibited a ζ of approximately −4 mV, consistent with slightly negatively charged Pluronic systems. Upon drug loading, the zeta potential increased and the average micelle diameter also rose (Fig. 4A),^45,74^ consistent with entrapment of doxorubicin.^75^ The micelle size remained stable after immersion in PBS at both room temperature and 37 °C (Fig. 4B), supporting the suitability of these drug-loaded micelles for storage at room temperature and subsequent use in hydrogel fabrication and use at physiological temperatures.

We then evaluated the release kinetics of doxorubicin from five hydrogel formulations: (i) neat gelatin hydrogels directly loaded with doxorubicin GEL/DOXO, (ii) gelatin hydrogels with physically incorporated Pl micelles and directly added doxorubicin (GEL/DOXO_Pl), (iii) gelatin hydrogels with chemically crosslinked Pl_Nb micelles and directly added doxorubicin (GEL/DOXO_Pl_Nb), (iv) gelatin hydrogels containing physically embedded doxorubicin-loaded Pl micelles (GEL_Pl/DOXO), and gelatin hydrogels containing chemically crosslinked doxorubicin-loaded Pl_Nb micelles (GEL_Pl_Nb/DOXO). All formulations exhibited cumulative release of doxorubicin over time (Fig. 4C). Release was significantly faster for hydrogels in which doxorubicin was directly incorporated into the gelatin matrix (GEL/DOXO, GEL/DOXO_Pl, and GEL/DOXO_Pl_Nb). In contrast, hydrogels containing micelles pre-loaded with doxorubicin (GEL_Pl/DOXO and GEL_Pl_Nb/DOXO) showed slower, sustained release profiles, consistent with the controlled release behaviour conferred by micellar encapsulation.^76^ We hypothesize that diffusion is the main release mechanism: in GEL/DOXO, doxorubicin freely diffuses through the hydrogel, while in micelle-containing hydrogels, the drug must first exit the micelles before migrating through the network, slowing release, as previously reported for micelle-loaded.^77,78^

Finally, we tested the bioactivity of the released doxorubicin using a cancer cell line model (Fig. 4D).^79^ In all control conditions lacking doxorubicin, cell viability remained above 70%, demonstrating that the hydrogels and micelles alone were non-toxic. In contrast, doxorubicin-loaded micelles (Pl/DOXO, Pl_Nb/DOXO) and hydrogels (GEL/DOXO, GEL_Pl/DOXO, and GEL_Pl_Nb/DOXO) significantly reduced cell viability, confirming the successful release of bioactive drug and the preservation of its cytotoxic function.^80^ To further assess the impact of slowed drug release, we cultured cells with eluates obtained after 6 h of immersion of DOXO-loaded samples (GEL, GEL_Pl, and GEL_Pl_Nb), a time point at which GEL released significantly more DOXO than GEL_Pl and GEL_Pl_Nb (*p* < 0.05). Cell viability in eluates from GEL_Pl and GEL_Pl_Nb samples was higher than that from GEL (Fig. S11), indicating that the incorporation of DOXO within micelles prior to hydrogel formation effectively reduced the immediate drug release and consequently modulated the biological response.

## 4. Conclusion

We present a modular strategy to tailor composite hydrogels by integrating Pluronic® F127 micelles either physically or *via* covalent incorporation using norbornene-functionalised Pluronic (Pl_Nb). Pl_Nb synthesis achieved near-complete functionalisation, yielding stable, cytocompatible, and thermo-responsive micelles with efficient drug loading. In gelatin-norbornene hydrogels used as the main polymer network in the hydrogel, the mode of micelle incorporation dictated network mechanics and dynamics: chemical crosslinking conferred temperature-dependent behaviour and enhanced stress relaxation. All hydrogels were cytocompatible, and both physical and chemical incorporation modes enabled sustained release of bioactive doxorubicin compared to direct loading. This micelle–hydrogel coupling offers a versatile design strategy for drug delivery and regenerative medicine.

## Author contributions

N. C. N.: conceptualization, data curation, formal analysis, investigation, methodology, project administration, supervision, validation, writing – original draft. H. S.: data curation, formal analysis, investigation, methodology, validation, writing – review & editing. A. C.: conceptualization, funding acquisition, project administration, resources, supervision, writing – review & editing.

## Conflicts of interest

The authors declare no conflicts of interest related to this work.

## Supplementary Material

BM-014-D5BM01434D-s001

## Data Availability

The data supporting this article have been included as part of the supplementary information (SI). Supplementary information: MALDI-TOF spectra, qualitative images of the thermo-responsive properties of the Pluronic solutions, ^1^H NMR spectrum of gelatin_norbornene, qualitative images of crosslinked hydrogels, fold change of the rheological properties of hydrogels during temperature sweeps, micelle size at different temperatures, the indentation modulus of the hydrogels, the relaxation time and plateau during stress relaxation tests, the average size and polydispersity index of the micelles in solution, and the cytotoxicity of micelles loaded with different drug concentrations. See DOI: https://doi.org/10.1039/d5bm01434d. Other data supporting the results of this study can be provided upon request from the corresponding author.
