# Supplementary material for: Tailoring composite hydrogel performance via controlled integration of norbornene-functionalised Pluronic micelles
Source: Biomater Sci. 2025 Dec 3;14(2):518–30. doi: 10.1039/d5bm01434d (PMC12679351; doi:10.1039/d5bm01434d)
Supplement: BM-014-D5BM01434D-s001 [file BM-014-D5BM01434D-s001.pdf]

Supplementary Information (SI) for Biomaterials Science.
This journal is © The Royal Society of Chemistry 2025

SUPPLEMENTARY INFORMATION

Tailoring Composite Hydrogel Performance via Controlled Integration of Norbornene-Functionalised Pluronic Micelles.

AUTHORS

Nicola Contessi Negrini[1, 2], Hongning Sun[1, 2], Adam D Celiz[1, 2, *]

AFFILIATIONS

[1] Department of Bioengineering, Imperial College London, London (United Kingdom)

[2] The Francis Crick Institute, London (United Kingdom)

*** CORRESPONDENCE**

a.celiz@imperial.ac.uk

Sir Uren Hub, Imperial College London

86 Wood Lane, W12 0BZ

London, United Kingdom

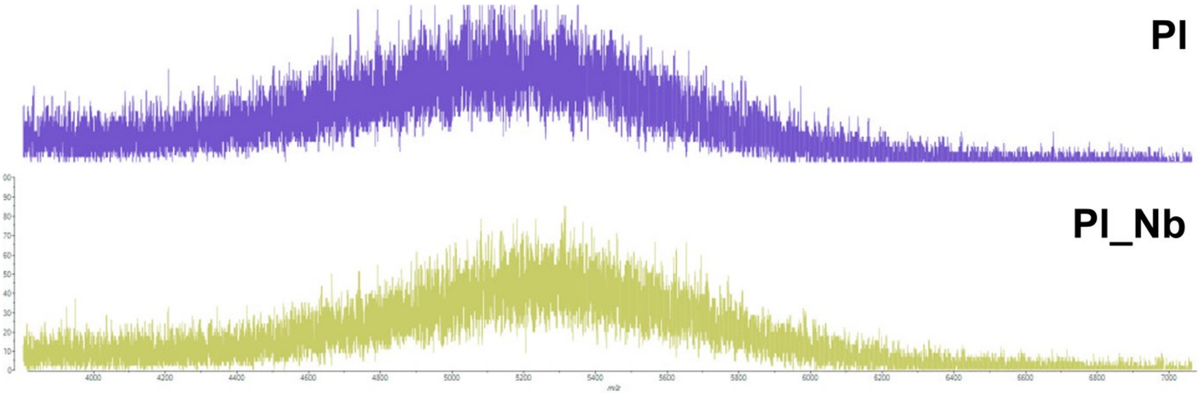

Figure S1. MALDI TOF spectra of PI and PI_Nb showing the specific peaks for each m/z.

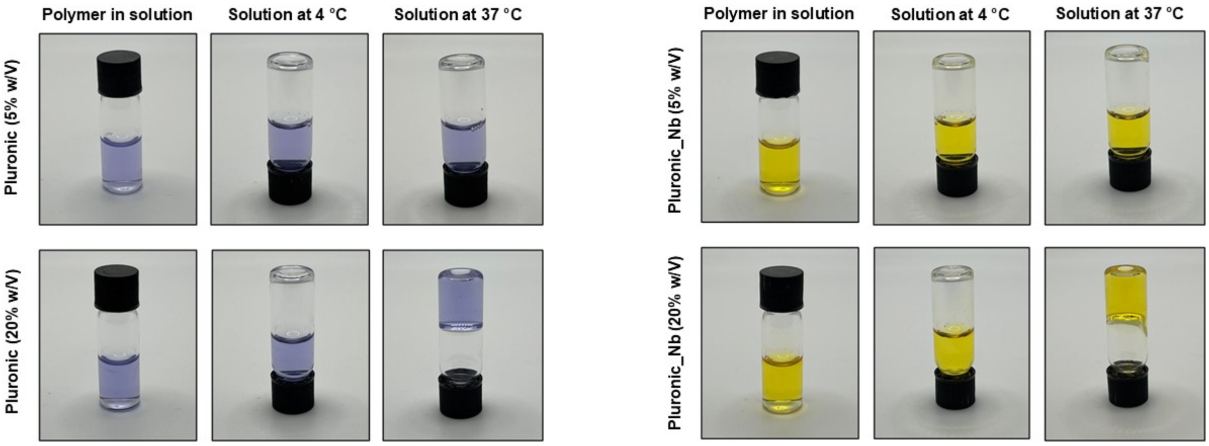

Figure S2. Representative images of inversion tests of Pluronic (PI) and Pluronic_Norbonene (PI_Nb) dissolved in water and stored at 4 °C and 20 °C (a dye was added for visualisation). The inversion tests show that both PI and PI_Nb 5% w/V are liquid at 4 °C. When the polymer concentration increases to 20% w/V, both PI and PI_Nb are in liquid form at 4 °C (i.e., $T < T_{sol\text{-}gel}$) while they undergo gelation at 20 °C (i.e., $T > T_{sol\text{-}gel}$).

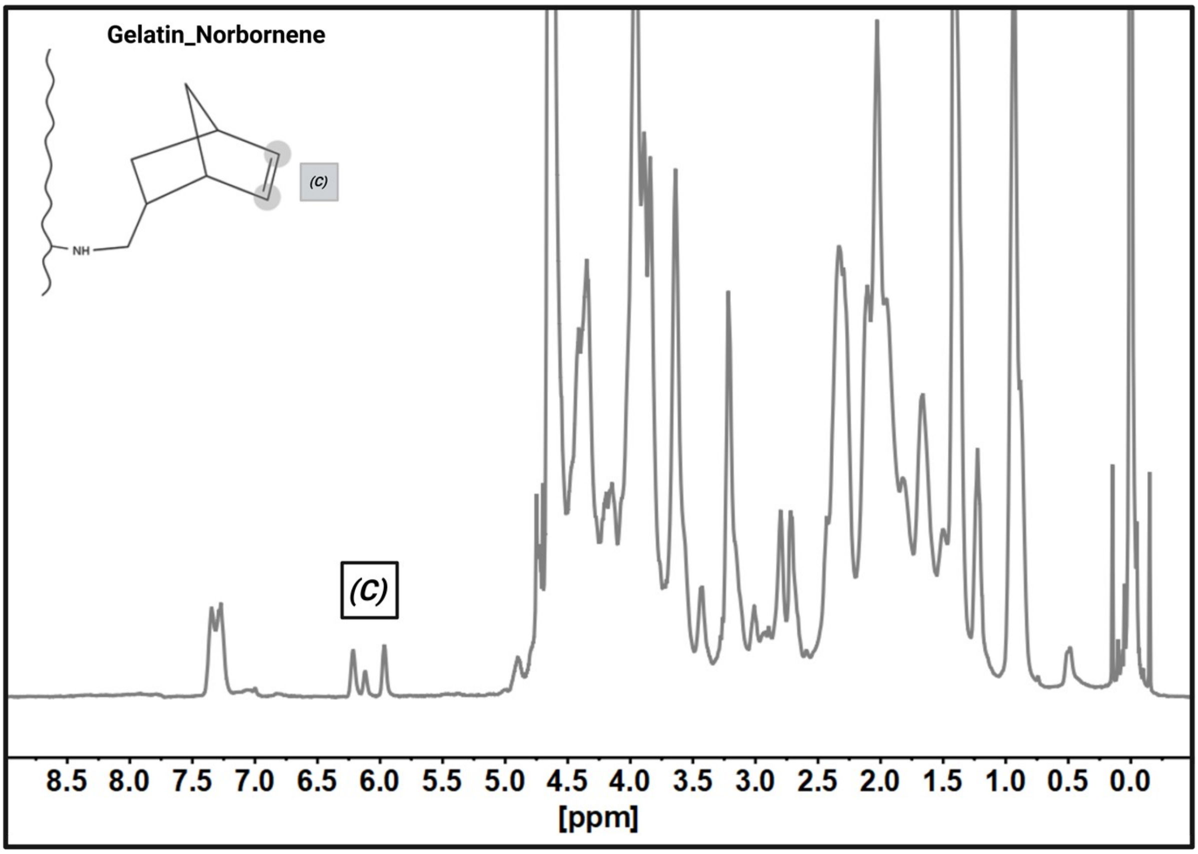

Figure S3. Representative ^{1}H NMR spectrum of Gelatin_Norbornene used as the polymer backbone for the preparation of the hydrogels.

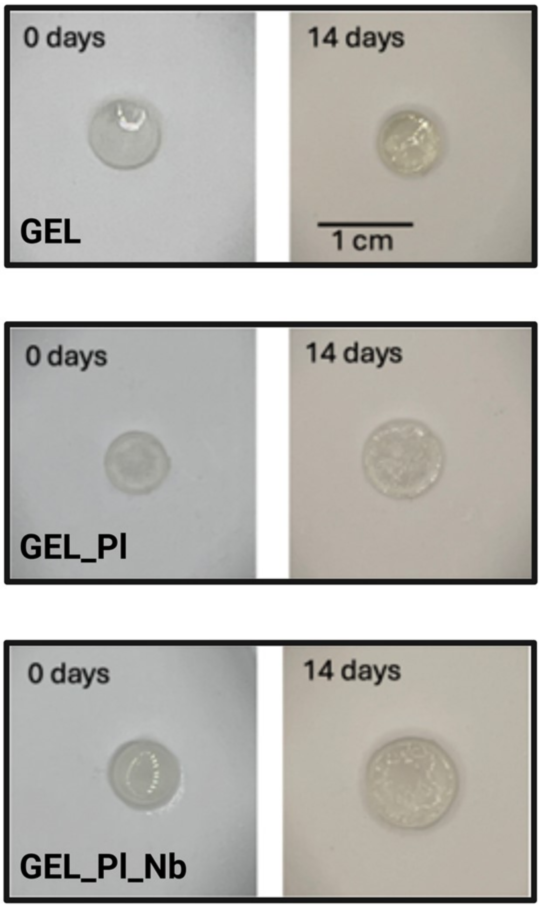

Figure S4. Representative images of freshly prepared hydrogels and hydrogels after 14 days of swelling in PBS at 37 °C (scale bar = 1 cm).

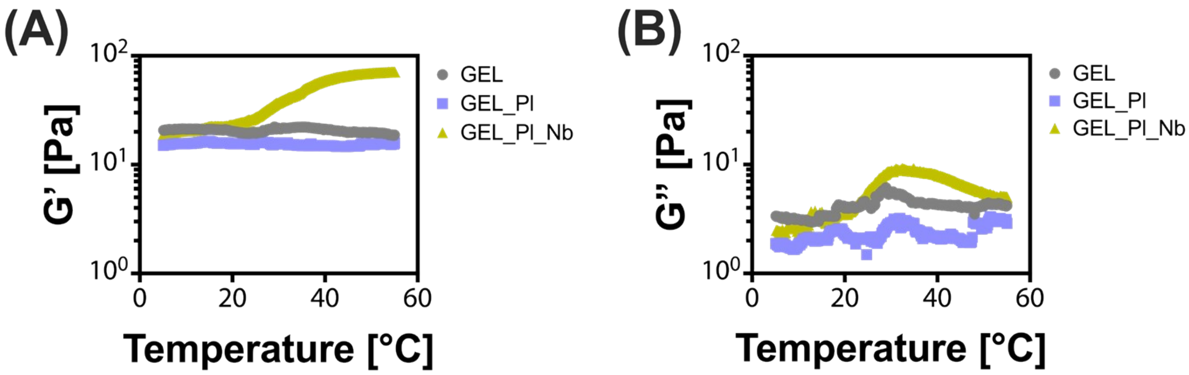

Figure S5. Detail of the thermo-responsive properties of the crosslinked hydrogels: **(A)** Storage Modulus, G', and **(B)** Loss Modulus, G".

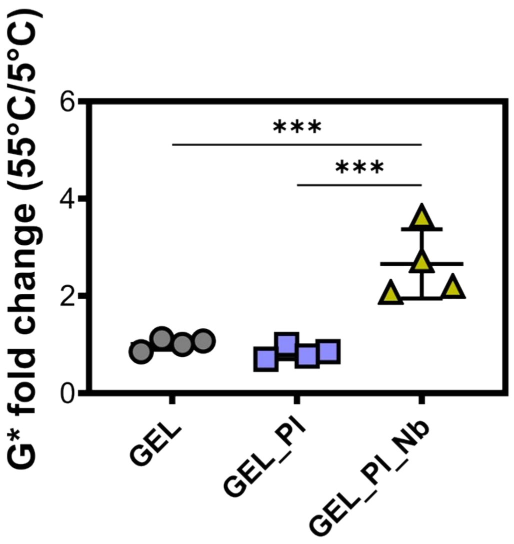

Figure S6. Fold change of the Complex Modulus G* of GEL, GEL_PI, and GEL_PI hydrogels from 5 °C to 55 °C. (*n* = 4)

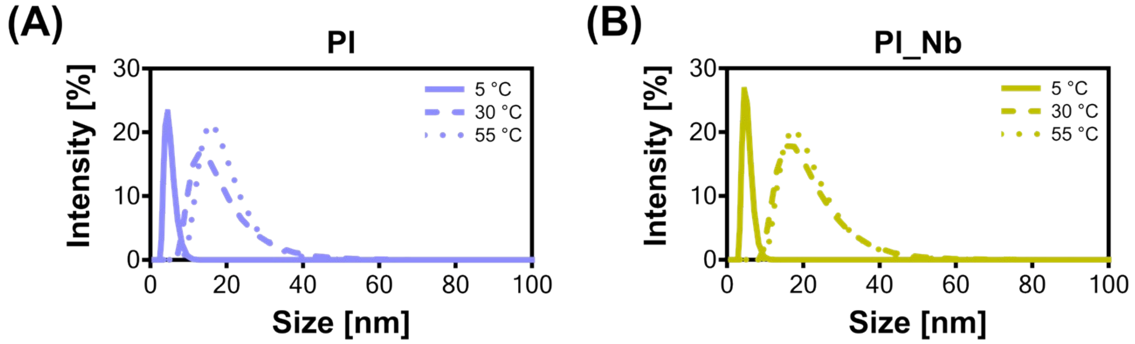

Figure S7. Size distribution of **(A)** PI and **(B)** PI_Nb micelles prepared at 2.5% w/v and tested at different temperatures (5, 30, and 55 °C).

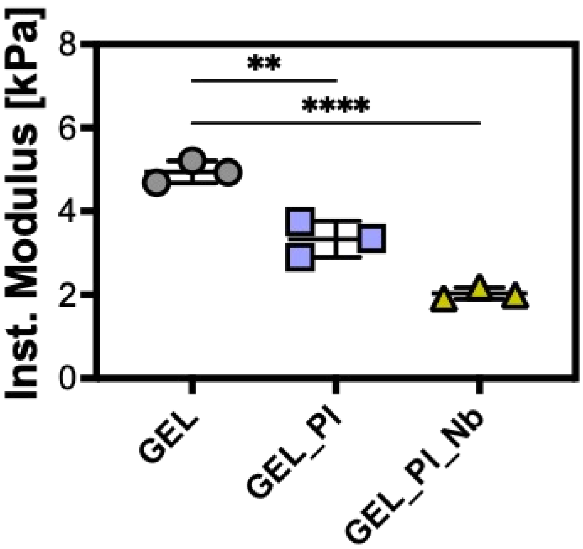

Figure S8. Indentation modulus (instantaneous modulus) for GEL, GEL_PI, and GEL_PI_Nb hydrogel formulations (*n* = 3).

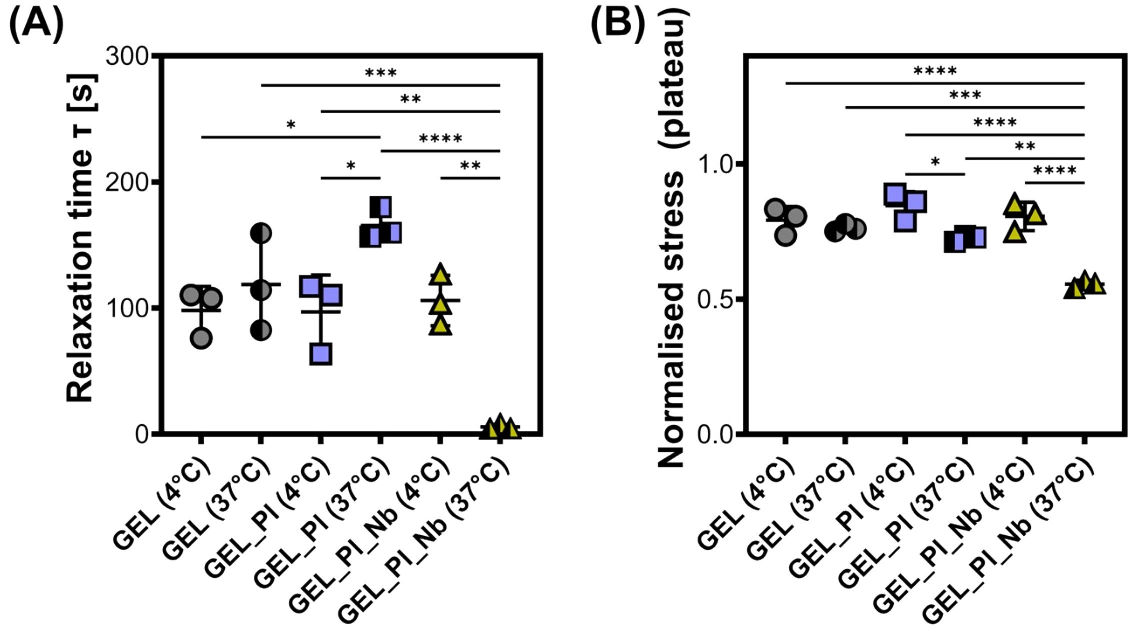

Figure S9. Stress relaxation of GEL, GEL_PI, and GEL_PI_Nb hydrogels tested via rheology at 4 and 37 °C: **(A)** relaxation time τ and **(B)** normalised stress at relaxation plateau (*n* = 3).

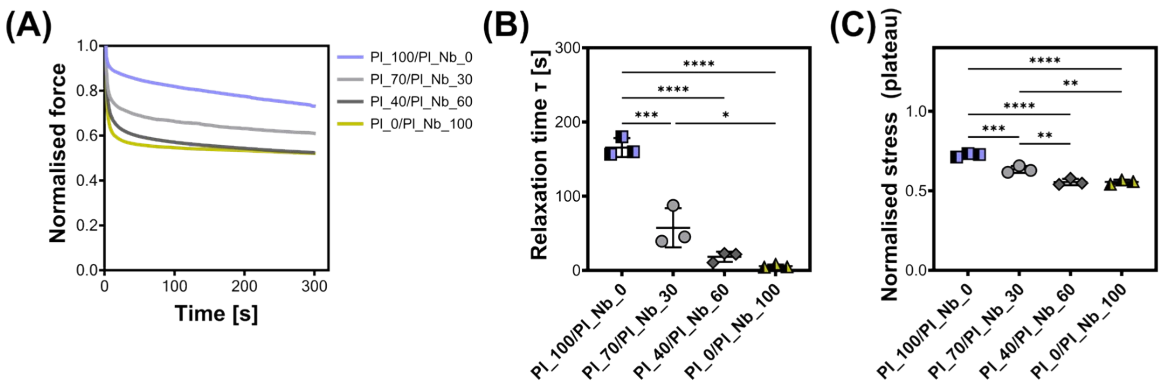

Figure S10. Stress relaxation of hydrogels prepared with different ratios of PI and PI_Nb micelles (from 100% PI and 0% PI_Nb, PI_100/PI_Nb_0, to 0% PI and 100% PI_Nb). **(A)** Representative normalised force in time during the stress relaxation test, **(B)** relaxation time τ, and **(C)** normalised stress at relaxation plateau (*n* = 3).

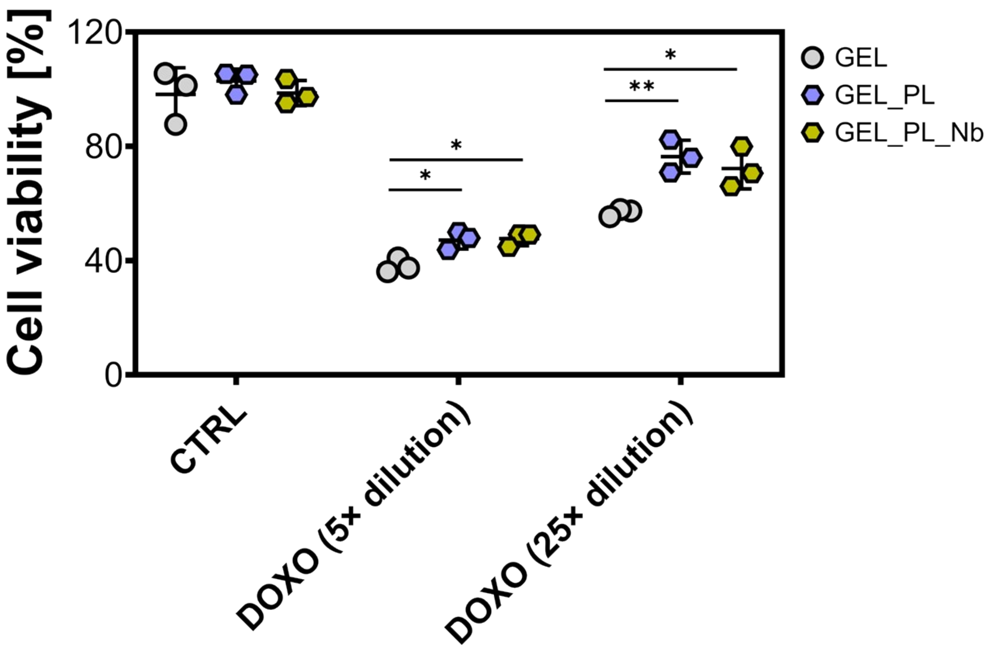

Figure S11. Viability of B16F10 cells cultured in supernatants collected after 6 h of immersion of DOXO-loaded samples (GEL, GEL_PI, GEL_PI_Nb), diluted 5× and 25× in culture medium. Non-loaded samples (CTRL) were used as controls (*n* = 3).

Table S1. Average size and polydispersity index (PDI) of Pl_Nb micelles prepared with different polymer concentrations (2.5., 5, 10, and 15% w/v).

	**Average Size [nm]**	**PDI**
**Pl_Nb_2.5**	22.16 ± 0.21	0.11 ± 0.07
**Pl_Nb_5**	26.14 ± 0.80	0.22 ± 0.09
**Pl_Nb_10**	18.66 ± 0.48	0.24 ± 0.02
**Pl_Nb_15**	18.56 ± 2.88	0.39 ± 0.10
